# The impact of fentanyl on state- and county-level psychostimulant and cocaine overdose death rates by race in Ohio from 2010 to 2020: a time series and spatiotemporal analysis

**DOI:** 10.1186/s12954-024-00936-9

**Published:** 2024-01-17

**Authors:** Angela T. Estadt, Brian N. White, JaNelle M. Ricks, Kathryn E. Lancaster, Staci Hepler, William C. Miller, David Kline

**Affiliations:** 1 Division of Epidemiology, College of Public Health, The Ohio State University, Columbus, USA; 2 Division of Public Health Sciences, Department of Biostatistics and Data Science, Wake Forest University School of Medicine, Winston-Salem, USA; 3 Division of Health Behavior and Health Promotion, College of Public Health, The Ohio State University, Columbus, USA; 4 Division of Public Health Sciences, Department of Implementation Science, Wake Forest University School of Medicine, Winston-Salem, USA; 5 Department of Statistical Sciences, Wake Forest University, Winston-Salem, USA; 6 Department of Epidemiology, Gillings School of Global Public Health, University of North Carolina at Chapel Hill, Chapel Hill, USA

**Keywords:** Psychostimulants, Cocaine, Fentanyl, Overdose, Spatiotemporal, Time series

## Abstract

**Background:**

Over the past decade in the USA, increases in overdose rates of cocaine and psychostimulants with opioids were highest among Black, compared to White, populations. Whether fentanyl has contributed to the rise in cocaine and psychostimulant overdoses in Ohio is unknown. We sought to measure the impact of fentanyl on cocaine and psychostimulant overdose death rates by race in Ohio.

**Methods:**

We conducted time series and spatiotemporal analyses using data from the Ohio Public Health Information Warehouse. Primary outcomes were state- and county-level overdose death rates from 2010 to 2020 for Black and White populations. Measures of interest were overdoses consisting of four drug involvement classes: (1) all cocaine overdoses, (2) cocaine overdoses not involving fentanyl, (3) all psychostimulant overdoses, and (4) psychostimulant overdoses not involving fentanyl. We fit a time series model of log standardized mortality ratios (SMRs) using a Bayesian generalized linear mixed model to estimate posterior median rate ratios (RR). We conducted a spatiotemporal analysis by modeling the SMR for each drug class at the county level to characterize county-level variation over time.

**Results:**

In 2020, the greatest overdose rates involved cocaine among Black (24.8 deaths/100,000 people) and psychostimulants among White (10.1 deaths/100,000 people) populations. Annual mortality rate ratios were highest for psychostimulant-involved overdoses among Black (aRR = 1.71; 95% CI (1.43, 2.02)) and White (aRR = 1.60, 95% CI (1.39, 1.80)) populations. For cocaine not involving fentanyl, annual mortality rate ratios were similar among Black (aRR = 1.04; 95% CI (0.96,1.16)) and White (aRR = 1.02; 95% CI (0.87, 1.20)) populations. Within each drug category, change over time was similar for both racial groups. The spatial models highlighted county-level variation for all drug categories.

**Conclusions:**

Without the involvement of fentanyl, cocaine overdoses remained constant while psychostimulant overdoses increased. Tailored harm reduction approaches, such as distribution of fentanyl test strips and the removal of punitive laws that influence decisions to contact emergency services, are the first steps to reduce cocaine overdose rates involving fentanyl among urban populations in Ohio. In parallel, harm reduction policies to address the increase in psychostimulant overdoses are warranted.

**Supplementary Information:**

The online version contains supplementary material available at 10.1186/s12954-024-00936-9.

## Introduction

Cocaine and psychostimulant overdose death rates involving fentanyl have risen dramatically in the USA [1]. From 2010 to 2020, the overdose death rates involving psychostimulants in the USA increased by 1000%, followed by cocaine at nearly 400%. The increase in overdose death rates of these substances echoes the “fourth wave” of the opioid overdose epidemic, a wave defined by the use of cocaine or psychostimulants such as methamphetamine mixed with fentanyl [2]. This growth of fentanyl and stimulant overdoses coincides with increases in fentanyl overdoses due to intentional and unintentional co-use [3–7]. While overdose deaths involving opioids and psychostimulants or cocaine have climbed, psychostimulant and cocaine deaths without the involvement of opioids have also increased [1]. From 2010 to 2020, annual cocaine deaths without opioid involvement rose from 2669 to 4984 deaths. In the same time period, annual psychostimulant deaths without opioids increased from 1655 to 11,400 deaths [1]. While fentanyl’s impact on overdose death rates is clear, the rate of change in annual overdose deaths attributable to psychostimulants and cocaine, not involving fentanyl, is unknown.

Trends in US opioid overdose mortality rates with cocaine and psychostimulants vary by race and geography [8]. Historically, cocaine was most prevalent among urban Black Americans, while psychostimulants such as methamphetamine were common among rural White Americans. But overdose rates involving cocaine with opioids grew nearly sixfold among Black Americans from 2007 to 2019; in contrast, cocaine overdoses with opioids grew only twofold among White Americans [8]. Similarly, methamphetamine with opioid overdose rates rose 160-fold among Black Americans compared to 32-fold among White Americans. Sociocultural contexts such as stereotyping [9], historically unethical medical treatment from clinicians [10], and the criminalization of drug use [11, 12] contribute to racial disparities of substance use patterns. Black Americans are less likely to have access to diverse substance use treatment services or engage in drug use treatment compared to White Americans [13, 14]. Opioid mortality rates involving psychostimulants or cocaine have increased the most steeply in the Midwest region of the USA, where substance use stigma and harm reduction and treatment service disparities persist [8, 15–17]. Due to these variations in overdose mortality trends by race and geography, finer resolution trends with racial subgroups are needed to determine locations and targeted populations for tailored harm reduction and treatment services.

Ohio, a state located in the Midwest region, was home to “pill mills” (i.e., medical clinics that overprescribe opioids) [18], economic downturns in rural communities [18], and an established drug trafficking network [19] early in the opioid epidemic. In parallel, Ohio’s Bureau of Criminal Investigation graphed rising rates of psychostimulant overdose deaths, involving and not involving fentanyl, in the state from 2013 to 2017 [20]. Furthermore, cocaine overdose death rates involving fentanyl appeared to increase during the same time period. Sociocultural circumstances that led to these increases likely include an effort to moderate the effects of opioid use, reduced purchasing costs, and the ability to remain alert to avoid becoming the victim of a crime [4, 6]. These characteristics provide unique geographical and sociocultural circumstances for the psychostimulant, cocaine, and opioid epidemics. Furthermore, mortality rates may differ by location and race due to Ohio’s diverse urban and rural landscape which consists of 8 unique racially diverse urban counties surrounded by 80 racially homogeneous rural counties [21]. Compared to other US states, Ohio and West Virginia showed particularly sharp increases in trends of both opioid overdose death rates involving psychostimulants and cocaine, while other states tended to concentrate on either cocaine or psychostimulants and opioids [22]. Although mortality rates of psychostimulants or cocaine with opioids have risen, whether mortality rates of psychostimulants and cocaine not involving fentanyl have also risen in unclear. To inform and tailor harm reduction efforts, we determined the contribution of fentanyl to cocaine and psychostimulant overdose rates by race and location.

Our primary objective was to analyze trends in Ohio of four classes of overdose death rates: (1) all cocaine overdoses, (2) all cocaine overdoses not involving fentanyl, (3) all psychostimulant overdoses, and (4) all psychostimulant overdoses not involving fentanyl. Our secondary objectives were to determine whether temporal trends in overdose deaths varied by race, determine whether there was variation in overdose death rates at the county-level, and to determine whether overdose rates varied over time by location and race. We hypothesized that (1) the rise in psychostimulant and cocaine overdose deaths in Ohio would be solely driven be fentanyl and vary by location and (2) increases in cocaine-involved and psychostimulant-involved overdose rates would be higher among Ohio’s Black population, reflecting overdose trends among Black Americans in the USA [8].

## Methods

### Study design

We conducted a state-level time series analysis and a county-level spatiotemporal analysis of stimulant (i.e., psychostimulant and cocaine) overdose deaths in Ohio from 2010 to 2020. For this study, we define overdoses as deaths classified as unintentional drug poisonings. We stratified stimulant overdose death rates by Black and White populations, and within each racial group, we considered four classes of drug involvement (all psychostimulant and cocaine overdoses and psychostimulant and cocaine overdoses not involving fentanyl) using data from the Ohio Public Health Information Warehouse [23]. The Ohio Public Health Information Warehouse “stores large volumes of vital data to support ongoing activities such as surveillance, assessments, grant writing, and evaluations” [24]. While race data were available for American Indian or Alaskan Native, Asian or Pacific Islander, and Other, we excluded these groups due to small overdose death counts by year and county over the time period.

The Ohio Public Information Warehouse defined overdose deaths using ICD-10 codes reported on Ohio death certificates. Psychostimulant overdoses were classified using the ICD-10 code T43.6, or “psychostimulants with abuse potential, including methamphetamine” and cocaine overdose deaths using the ICD-10 code T40.5. Fentanyl and fentanyl analogue-related deaths were defined as, “a positive mention of select text strings in the death certificate text.”

### Time series analysis of stimulant overdoses in Ohio, 2010–2020

We conducted state-level time series analyses of standardized mortality rates for each of the four classes based on simulant type and fentanyl involvement in Ohio from 2010 to 2020. We used indirect standardization and the 2010 Ohio population from the Ohio Public Health Information Warehouse to calculate the expected number of age-adjusted overdose deaths, using counts by age categories, for each substance group: cocaine and psychostimulants. To assess the contribution of fentanyl, we estimated trends for stimulant-involved overdose deaths that do not involve fentanyl by race and compared the trends for stimulant-involved deaths not involving fentanyl to those for all stimulant-involved deaths. To compare the expected and actual overdose death rates over time, we calculated standardized mortality ratios (SMRs) using Bayesian generalized linear mixed models. The models included fixed effects for race (Black/White), time (year), and a race–time interaction. The model also included a race-specific temporal autoregressive (AR) (1) random effect. The inclusion of these effects allowed us to determine standardized mortality rates over time by race and to compare race groups for each substance. Posterior medians and 95% credible intervals were computed for each comparison of interest. Full statistical details for time series and spatiotemporal analyses are included in Additional file 1.

### Spatiotemporal analysis

We conducted a county-level spatiotemporal analysis to determine the variation of overdose rates in Ohio from 2010 to 2020. We modeled the annual standardized mortality rate for each overdose pattern by adapting a Bayesian multivariate spatial rates mixed effects Poisson regression model to estimate county-level SMRs [25]. As in the time series analysis, we include fixed effects for race and time. We also include temporal random effects to capture race- and county-specific temporal variation and a shared spatiotemporal random effect to account for county-specific variation common to both race groups and spatial correlation.

## Results

### Crude analysis

From 2010 to 2020, Black Ohioans experienced 2200 overdose deaths involving cocaine and 193 overdose deaths involving psychostimulants. In contrast, White Ohioans experienced 6324 overdose deaths involving cocaine and 3257 deaths involving psychostimulants. Over this period, the rate of overdoses involving psychostimulants increased 6.9 times among Black Ohioans (from 0.07 deaths/100,000 people in 2010 to 4.8 deaths/100,000 people in 2020); the rate of overdoses for cocaine increased 6.1 times in this group (from 4.1 deaths/100,000 people to 24.8 deaths/100,000 people). Over the same period, the rate of overdoses involving psychostimulants increased 10 times among White Ohioans (from 0.9 deaths/100,000 people in 2010 to 10.1 deaths/100,000 people in 2020); the rate of overdoses for cocaine increased 6.8 times (from 1.5 deaths/100,000 people to 8.4 deaths/100,000 people). For context, the reported rate of overdoses involving fentanyl increased 46 times among White Ohioans (from 0.8/100,000 people in 2010 to 37.3/100,00 people in 2020) and, among Black Ohioans, from 3 fentanyl-involved overdoses in 2010 to 696 overdoses in 2020 [23]. Due to the small number of fentanyl-involved overdoses in 2010 among Black Ohioans, the age-adjusted overdose rate is considered unreliable and is therefore not reported here.

**Fig. 1:**
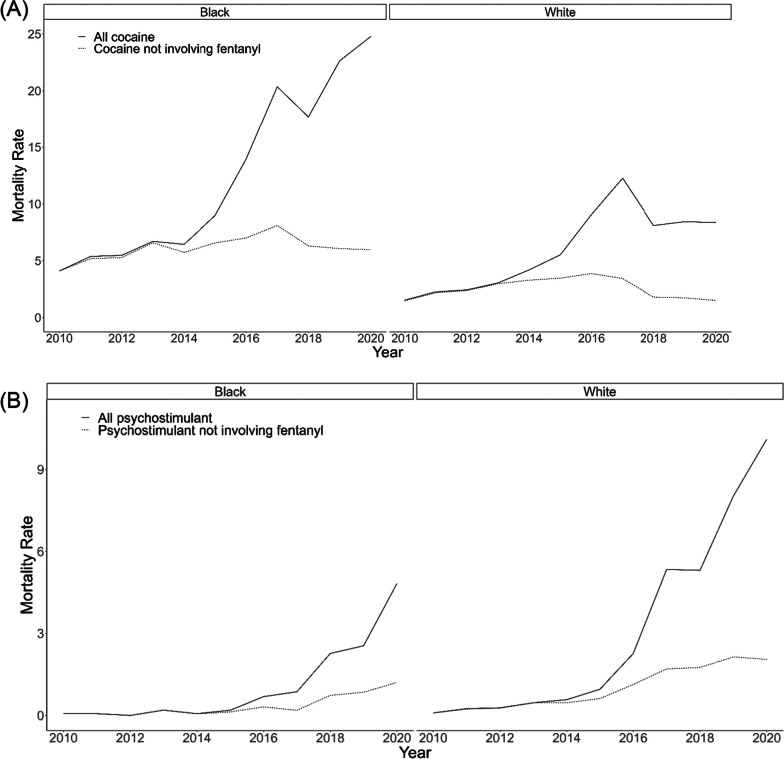
Ohio statewide overdose death rate (per 100,000 people) from 2010 to 2020 by race and substance overdose patterns. **A** Rate of cocaine-involved overdoses stratified by race (Black/White). The solid line indicates death rates for all overdoses involving cocaine, while the dotted line indicates cocaine-involved overdoses not involving fentanyl. Notably, cocaine overdose rates increased sharply in 2013 and 2014. **B** Rate of psychostimulant-involved overdoses stratified by race (Black/White). The solid line indicates death rates for all overdoses involving psychostimulants, while the dotted line indicates psychostimulant-involved overdoses not involving fentanyl. Sharp increases in psychostimulant overdose rates, involving and not involving fentanyl, began in 2014

Cocaine overdose death rates were highest among Black Ohioans in the beginning of the study period, while psychostimulant overdose death rates were small but higher for White compared to Black Ohioans in 2010 (Fig. 1). Increases among all cocaine and psychostimulant overdoses indicate fentanyl likely entered drug supplies near 2013.

### State-level time series analysis

**Fig. 2:**
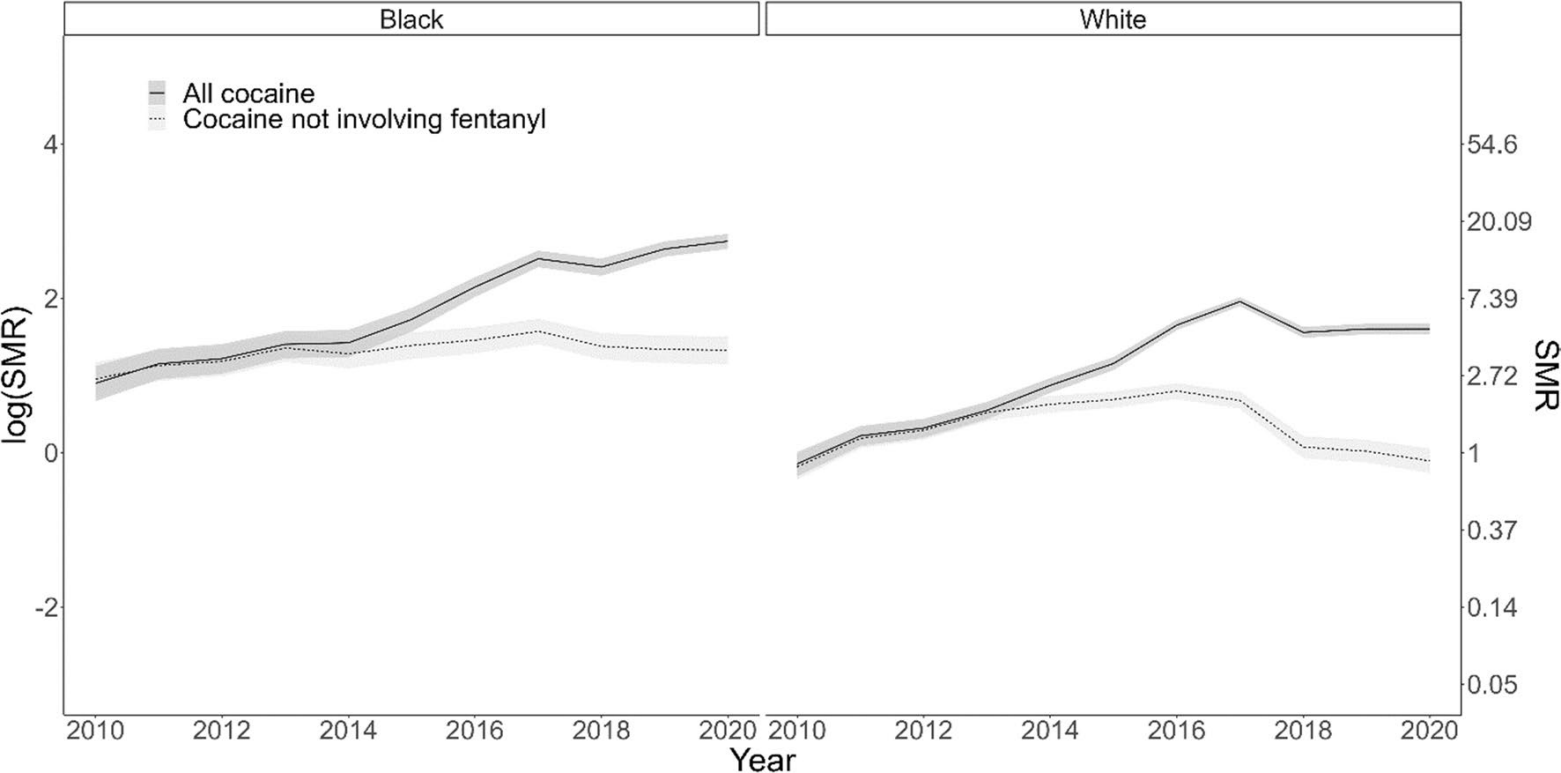
Standardized mortality rates with 95% credible intervals for overdoses involving cocaine, by race, Ohio 2010–2020. The standardized mortality rates of cocaine-involved overdoses over time on the log scale, by race (Black/White) using the 2010 cocaine-involved overdose rate as the standard. For both races, the solid line of standardized mortality rates of all cocaine-involved overdoses was significantly higher than cocaine standardized mortality rates not involving fentanyl beginning in 2013/2014

At the state level, overdose deaths involving stimulants increased over the past decade among both Black and White racial groups. Relative to the expected number of deaths based on 2010 mortality rates, death rates involving cocaine were 15.5 (95% credible interval (CrI) 14.0, 17.0) times higher in Black residents and 5.0 (95% CrI 4.6, 5.3) times higher in White residents in 2020 (Fig. 2). Rates of cocaine-involved overdose deaths in Black residents were 2.11 (95% CrI 1.04, 4.64) times the rates in White residents in 2010. The average annual percentage change in overdose death rates involving cocaine was an increase of 21% (RR 1.21, 95% CrI 1.11, 1.31) in Black residents and 20% (RR 1.20, 95% CrI 1.06, 1.36) in White residents. However, we did not have evidence that the annual change differed by racial group, as the annual increase in White residents was 0.99 (95% CrI 0.86, 1.15) times the increase in Black residents.

To assess the impact of fentanyl on cocaine-involved overdose deaths, we describe trends after removing deaths involving both cocaine and fentanyl. For cocaine-involved deaths not involving fentanyl, death rates in Black residents are 2.41 (95% CrI 0.95, 5.84) times those of White residents in 2010. But neither Black (RR 1.04, 95% CrI 0.96, 1.16) or White (RR 1.02, 95% CrI 0.87, 1.20) residents displayed meaningful changes over time, suggesting that much of the increase in cocaine-involved deaths is attributable to fentanyl.

**Fig. 3:**
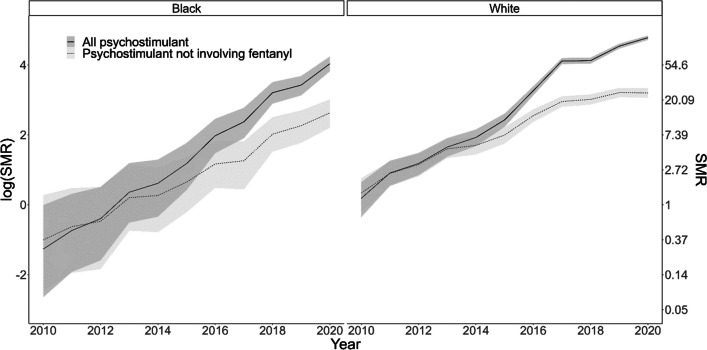
Standardized mortality rates with 95% credible intervals for overdoses involving psychostimulants, by race, Ohio 2010–2020. The standardized mortality rates of psychostimulant-involved overdoses over time on the log scale, by race (Black/White), using the 2010 psychostimulant-involved overdose rate as the standard. On the left, all psychostimulant-involved standardized mortality rates (solid line) rose significantly more than psychostimulant overdoses not involving fentanyl in 2017 for Black Ohioans. In contrast, all psychostimulant-involved standardized mortality rates (solid line) rose significantly more than psychostimulant overdoses not involving fentanyl earlier, in 2015, for White Ohioans

Death rates in 2020 involving psychostimulants were 56.4 (95% CrI 45.4, 69.5) times higher in Black residents and 118.8 (95% CrI 111.4, 126.4) times higher in White residents compared to what would be expected based on rates in 2010 (Fig. 3). Rates of psychostimulant-involved overdose deaths in Black residents were 0.20 (95% CrI 0.05, 0.69) times the rate in White residents in 2010. That is, psychostimulant-involved death rates were five times greater in White residents than in Black residents. The average annual percentage change in overdose death rates involving psychostimulants was an increase of 71% (RR 1.71, 95% CrI 1.43, 2.02) in Black residents and 60% (RR 1.60, 95% CrI 1.39, 1.80) in White residents. The annual change did not differ meaningfully by racial group, as the annual increase in White residents was 0.94 (95% CrI 0.75, 1.16) times the increase in Black residents.

We also examined psychostimulant-involved overdose deaths after removing deaths that involved fentanyl. For psychostimulant-involved deaths not involving fentanyl, rates in Black residents are 0.19 (95% CrI 0.04, 0.73) times those in White residents. That is, death rates were 5.3 times greater in White than Black residents. The average annual percent change in overdose death rates involving psychostimulants not involving fentanyl was an increase of 44% (RR 1.44, 95% CrI 1.20, 1.73) in Black residents and 34% (RR 1.34, 95% CrI 1.16, 1.53) in White residents (Table 1). We had little evidence that the change over time differed by race group as the change in White residents was 0.93 times the change in Black residents (RR 0.93, 95% CrI 0.73, 1.16), suggesting that psychostimulant overdose death rates increased despite fentanyl; fentanyl appeared to have exacerbated this trend.

**Table 1:** Cocaine- and psychostimulant-involved overdose rate ratios by annual change and race, Ohio 2010–2020

	Time (1 year change)		Race Comparison	
	RR^1^	95% CrI^1^	RR^1^	95% CrI
All cocaine overdoses				
White	1.20*	(1.06, 1.36)	ref	–
Black	1.21*	(1.11, 1.31)	1.01	(0.87, 1.16)
All psychostimulant overdoses				
White	1.60*	(1.39, 1.80)	ref	–
Black	1.71*	(1.43, 2.02)	1.07	(0.87, 1.33)
Cocaine overdoses *not involving* fentanyl				
White	1.02	(0.87, 1.20)	ref	–
Black	1.04	(0.96, 1.15)	1.02	(0.85, 1.23)
Psychostimulant overdoses *not involving* fentanyl				
White	1.34*	(1.16, 1.53)	ref	–
Black	1.44*	(1.20, 1.73)	1.07	(0.86, 1.36)

^1^CrI = credible interval; RR = rate ratio

*Associated credible interval does not include the null value (“1”)

### County-level Spatiotemporal Analysis

We conducted a county-level analysis from 2010 to 2020 to further explore heterogeneity in overdose mortality. A county-level map of Ohio with referenced cities is available to readers in Additional file 2: Fig. S1. In general, similar temporal trends and relative rates of mortality between Black and White residents were observed within counties across the state (Additional file 2: Figs. S2–S5). That is, cocaine-involved overdose death rates were higher among Black residents, and psychostimulant-involved death rates were higher among White residents. Rates of overdose deaths involving both types of stimulants increased over time in counties across the state. Despite these shared trends, we observed regional heterogeneity in the rates of overdose deaths for the two drug groups. Since the temporal and racial patterns are similar across the state, we can most clearly identify the regions affected by each drug type by examining estimates of the term in the model capturing variability shared between the race groups. This term highlights counties where both race groups are above or below the 2010 expected death rate.

**Fig. 4:**
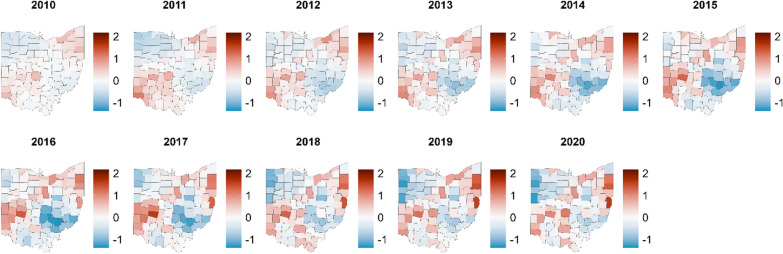
Choropleth maps of shared spatial component analysis for cocaine overdose deaths in Ohio, 2010–2020. Posterior estimates of counties with above average estimates of all cocaine-involved overdose deaths (red) and lower than average estimates (blue), by year, 2010–2020. The shared component reflects variation and trends common to both racial groups. Highest estimates are seen in urban counties such as those that include Akron, Cincinnati, Cleveland, Columbus, and Dayton

Cocaine-involved overdose deaths were highest for both Black and White Ohioans in southwest and northeast urban cities (Fig. 4). The posterior probabilities that the cocaine overdose deaths were higher than average were highest in these counties (Additional file 2: Fig. S6). These regions correspond to Cincinnati, Dayton, Akron, and Cleveland and reflect parts of the state that are more urban, racially diverse, have historically relied on manufacturing, and could be considered part of the Rust Belt. Lower rates are observed in the state’s more racially homogeneous rural and Appalachian eastern and southeastern regions. These areas are less than zero, indicating they tended to be below the 2010 expected number of deaths. Therefore, the aggregate increases over time observed in the state-level time series analyses appear to be concentrated in the urban, industrial regions of the state.

**Fig. 5:**
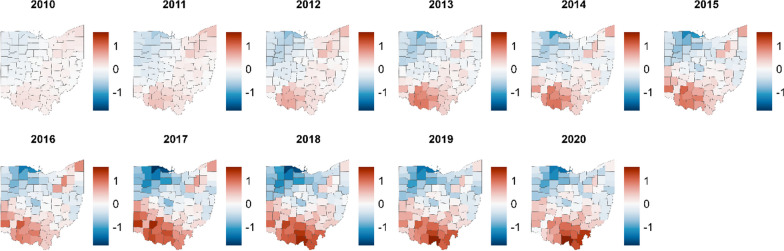
Choropleth maps of shared spatial component analysis for psychostimulant overdose deaths in Ohio, 2010 and 2020. Posterior estimates of counties with above average estimates of all psychostimulant-involved overdose deaths (red) and lower than average estimates (blue), by year, 2010–2020. The shared component reflects variation and trends common to both racial groups. The highest estimates are seen in southern Ohio, where the city of Portsmouth is located

Estimates of psychostimulant-involved overdose deaths were greatest in eastern Ohio for both race groups (Fig. 5). The posterior probability that these counties were above average is confirmed in Additional file 2: Fig. S7. While psychostimulant overdose rates increased across all counties, we see the highest rates in the southern portion of the state, particularly along the southern and southeastern borders. These counties are primarily rural and Appalachian, where the greatest increases in psychostimulant-involved overdose deaths have occurred. The spatiotemporal analysis produced similar posterior median overdose rate ratios to the time series analysis (Additional file 2: Table S1).

## Discussion

We conducted a state-level time series analysis to measure cocaine and psychostimulant overdose trends, unadjusted and adjusted for the involvement of fentanyl, in Ohio from 2010 through 2020. We found that psychostimulant overdose rates were exaggerated by and rising independently of fentanyl’s involvement. Furthermore, overdose rates vary over time, type of substance, and location in Ohio. In contrast, most of the rise in cocaine-related deaths appears to be related to fentanyl. Overall, Black Ohioans have higher cocaine overdose rates compared to White Ohioans, and this relationship is reversed for psychostimulant overdoses. Despite the differences in rates, all psychostimulant and cocaine overdose rates are rising in parallel between the races, i.e., overdose rates are not rising faster for one race compared to the other. We confirmed our findings using a county-level spatiotemporal analysis and observed that cocaine-involved overdose rates were highest in urban counties, while psychostimulant-involved rates were greatest in rural southern communities.

Psychostimulant overdose rates are rising both independently and with the involvement of fentanyl in Ohio. Our findings align with a state-level analysis of psychostimulant overdose rates that found an increase in overdose rates of psychostimulants not involving opioids in Ohio and the adjacent states of West Virginia, Kentucky, and Indiana [22]. To reduce overdose mortality rates, harm reduction efforts have been targeted toward opioid use [26], such as fentanyl, as overdoses can occur within minutes of use [27], opioid overdose rates are historically higher than psychostimulant and cocaine rates [28], and opioid overdose reversal medication exists to prevent fatal overdoses. In contrast, no overdose reversal medication is FDA-approved for psychostimulant or cocaine overdoses. Despite the lower mortality rates of methamphetamine and cocaine overdoses, these substances increase the risk of overdoses when used in combination with opioids by increasing the risk for cardiac arrest [29, 30]. These biological influences may help explain why methamphetamine and cocaine use is associated with increased risk for overdoses among people who use opioids [31, 32].

Disaggregating state-level overdose rates to smaller areas, in this case, counties, shows the varying distribution of psychostimulant and cocaine overdose mortality over the past decade. We found that cocaine overdose deaths, excluding fentanyl, were concentrated in northeast Ohio while psychostimulant overdose rates were highest in rural Appalachian Ohio. A spatiotemporal trend analysis of overdose rates in the USA showed that psychostimulant overdose rates were low across the country in 2012; by 2020, the highest psychostimulant overdose rates in the USA were clustered in counties in Appalachia, including southern Ohio [33]. Our findings align with these rates of psychostimulant overdoses in southern Ohio and support the notion that southern Ohio may have some of the highest psychostimulant overdose rates in the USA.

The annual rates of change of cocaine and psychostimulant overdoses did not vary between Black and White Ohioans from 2010 to 2020; however, Black Ohioans had a higher cocaine mortality rate compared to White Ohioans. Nationally, the prevalence of cocaine use has not increased more rapidly in Black populations compared to White populations [34] and non-Hispanic Black men and women had the highest rates of cocaine overdose deaths from 1999 to 2000 [35]. But for Black Ohioans who use cocaine, both increased fentanyl contamination and intentional co-use may have contributed to elevated overdose mortality. As states experience a new wave of increased fentanyl exposure concentrated in Black populations, the structural and systemic racism manifest in the design and provision of health care services limits access to corresponding harm reduction and evidence-based treatment services in highly segregated communities of color [36, 37]. For those able to access services, Black Americans also tend to have lower utilization rates, and less satisfaction with treatment compared to White counterparts [38, 39]. Overdose prevention efforts could be improved by increasing state and federal funding for local, culturally tailored services and encouraging people who primarily use cocaine to use fentanyl test strips.

We performed a spatial analysis to measure county-level differences in overdose rates and discovered similar conclusions to the state-level time series analysis, with geographical differences present regardless of race. Urban counties, or areas with larger proportions of Black Ohioans, had the highest increases in cocaine-involved overdose rates. In contrast, rural Appalachian counties, or areas with mostly White Ohioans, had the highest increases in psychostimulant-involved overdose rates. Aligning with previous research, psychostimulant overdose rates remained significantly elevated among White compared to Black populations in our study [40], while this relationship was reversed for cocaine overdoses [41]. Although White Ohioans remain primary users of psychostimulants, recently more Black Ohioans have identified as users [42]. This shift aligns with a disproportionate national increase in prevalence of psychostimulant substance use treatment admissions among Black Americans in recent years [43], a probable signal of change in usage. Recent expansion of the Ohio drug market—reflected in supply, costs, and buyer access—may influence cultural and community protections and perceptions in Black communities which have historically restricted the spread of psychostimulant use [44, 45]. To develop and implement effective illicit drug education, use prevention, and treatment programs, the patterns of and motivations for psychostimulant use across communities must be more clearly understood.

The public health implications of our findings echo other calls to increase treatment access for psychostimulant and cocaine use in concert with opioid use and reduce health disparities in harm reduction [2, 46–49]. In rural Illinois, emerging risk areas were less likely to have substance use resources, compared to areas with historically high overdose rates [50]. Similarly, in a national analysis, communities with historically poor health outcomes had more preventative resources available [51]. These findings align with Ohio: Substance use treatment centers are located in urban centers that are historically high intensity drug trafficking areas [19]. Our findings show the need for harm reduction and substance use treatment in their surrounding communities. Specifically, harm reduction measures such as fentanyl test strips [52], naloxone [53], and contingency management [54] can reduce morbidity associated with psychostimulant, cocaine, and fentanyl use. In the absence of medication assisted treatment for stimulants, a comprehensive harm reduction approach is needed. But the benefit of education programs as a supplement to overdose prevention efforts is unclear [55]. Informational campaigns on the physiological long-term effects of stimulant use [56, 57] have not been evaluated for effectiveness. Furthermore, harm reduction policies should be tailored to protect Black populations in Ohio. Despite current harm reduction policies, stigma and criminalization of substance use are compounded by racism [48]. Black people who use drugs are likely to avoid calling the police to report an overdose and do not trust Good Samaritan Laws due to the historical criminalization of drug use targeting of Black populations in the USA.

We did not examine any factors other than race, such as socioeconomic status and urbanicity, associated with locations with increased psychostimulant and cocaine overdose rates. Other factors than race have been associated with high opioid overdose rates, but other factors associated with psychostimulant and cocaine overdose rates are unclear. Future research should explore how socio-structural factors explain variation in overdose rates across space, time, and racial groups. As an ecological study, our findings are limited to the county level as other levels of aggregation may observe different associations, which may be of future research interest. Nonetheless, a rural risk environment analysis of southern Illinois found variation in fatal opioid overdose rates by zip code, and that geographical differences were attributed to higher poverty areas, presence of colleges, and income inequality [50]. In North Carolina, opioid overdose rates varied by space, time, and drug type: Western Appalachian counties had the highest prescription opioid overdose mortality over time, while heroin overdose rates were highest in urban counties [58]. In a national spatial analysis, opioid use disorder rates were influenced in some counties by race/ethnicity and mental health conditions of beneficiaries [59]. Our results are also limited by the accuracy of the cause(s) of death reported on the death certificates, of which they may have unintentionally underreported substances involved in an overdose death [60]. In Ohio from 2008 to 2010, 70% of drug intoxication deaths included a specified drug [61]. This underestimation may have caused nondifferential bias as the involvement of opioids may have been missed among cocaine or psychostimulant overdoses, or conversely among opioid overdoses. Furthermore, our study is limited in describing the change in overdose rates prior and during the initial months in 2020 of the COVID-19 pandemic. Overdose rates increased markedly during the pandemic, and for the first time, Black and Native American overdose rates surpassed that of White overdose rates [62], indicating racial disparities in Ohio may have amplified after our analysis period.

In our analyses, two stimulant stories emerged in Ohio. First, psychostimulant overdose rates increased, exaggerated by fentanyl’s influence. In contrast, rises in cocaine overdoses were primarily attributed to fentanyl. But when fentanyl is involved, cocaine overdose rates are markedly increased among both Black and White Ohioans. We demonstrated the evolution of growing psychostimulant overdose rates in rural southern Ohio. Similarly, we observed the expansion of cocaine-related overdose deaths into counties surrounding the highly urban areas of Dayton in the west and Cleveland and Akron in the northeast. These spatial differences suggest that tailored harm reduction measures are needed to address the high concentration of overdoses. Potential measures include overdose education campaigns, fentanyl testing strips [52] and naloxone [53] for those who use psychostimulants or cocaine, as well as contingency management programs [54]. Furthermore, policies that support community programs, such as drug courts and community rehabilitation centers, may encourage treatment initiation among people who use cocaine or psychostimulants.

### Supplementary Information

**Additional file 1**. Statistical Supplement.

**Additional file 2**. County-level maps of Ohio, including Black versus White rate ratio estimates of cocaine and psychostimulant-involved SMRs from 2010-2020, and a table of spatiotemporal cocaine and psychostimulant-involved overdose rate ratios by annual change and race.

## Data Availability

Data used in the analyses are publicly available on the Ohio Public Health Data Warehouse: https://odh.ohio.gov/explore-data-and-stats/interactive-applications/ohio-public-health-data-warehouse1. The Ohio Department of Health specifically disclaims responsibility for any analyses, interpretations, or conclusions.

## Abbreviations

AR: Autoregressive
aRR: Adjusted rate ratio
CrI: Credible interval
PM: Posterior median
RR: Rate ratio
SMR: Standardized mortality ratio
USA: United States of America
