# Supplementary material for: The impact of fentanyl on state- and county-level psychostimulant and cocaine overdose death rates by race in Ohio from 2010 to 2020: a time series and spatiotemporal analysis
Source: Harm Reduct J. 2024 Jan 17;21:13. doi: 10.1186/s12954-024-00936-9 (PMC10792830; doi:10.1186/s12954-024-00936-9)
Supplement: Supplementary file 1 — Additional file 1. Statistical Supplement. [file 12954_2024_936_MOESM1_ESM.pdf]

# Statistical supplement for “The impact of fentanyl on state and county-level psychostimulant and cocaine overdose death rates by race in Ohio from 2010-2020: a time series and spatio-temporal analysis”

Angela T Estadt
The Ohio State University, College of Public Health, Division of Epidemiology
E-mail: estadt.24@osu.edu

Brian N White
Wake Forest University School of Medicine, Division of Public Health Sciences, Department of Biostatistics and Data Science
E-mail: bnwhite@wakehealth.edu

JaNelle Ricks
The Ohio State University, College of Public Health, Division of Health Behavior and Health Promotion
E-mail: ricks.13@osu.edu

Kathryn E Lancaster
Wake Forest University School of Medicine, Division of Public Health Sciences, Department of Implementation Science
E-mail: klancast@wakehealth.edu

Staci Hepler
Wake Forest University, Department of Statistical Sciences
E-mail: heplersa@wfu.edu

William C Miller
The University of North Carolina at Chapel Hill, Gillings School of Global Public Health, Department of Epidemiology
E-mail: miller.8332@osu.edu

David Kline
Wake Forest University School of Medicine, Division of Public Health Sciences, Department of Biostatistics and Data Science
E-mail: dkline@wakehealth.edu

## 1 Statistical Models

For reference and completeness, the Bayesian hierarchical models are detailed here. These models were fit independently for each of the four drug categories present in the analysis using state-level and county-level overdose death count data obtained from the Ohio Public Health Information Warehouse. In the following exposition let $s$ indicate county ($s = 1, \ldots, 88$), $t$ year ($t = 2010, ..., 2020$) and $r$ race ($r = 0$ is White and $r = 1$ is Black).

### 1.1 Time-series model

A multivariate Poisson linear mixed effects model was used to perform the time-series analysis of the state-level overdose death count data. Assume that

$$Y_{tr}|\lambda_{tr} \overset{\text{ind}}{\sim} \text{Poisson}(\mathrm{E}_{tr}\lambda_{tr}) \tag{1}$$

where $Y_{tr}$ is number of overdose deaths in Ohio for year $t$ and race $r$ and $E_{tr}$ is the corresponding expected number of overdose deaths in Ohio for year $t$ and race $r$ based upon the age-specific overdose death rate in the 2010 Ohio population. In other words, $E_{tr}$ is the number of age-adjusted overdose deaths in Ohio for year $t$ and race $r$ obtained via indirect-standardization with the 2010 Ohio population serving as the reference population. This formulation assumes the expected overdose rates between racial groups are homogeneous.

$E_{tr}$ is defined as follows. First, denote the 2010 state-level overdose death rate for age group $i$ as $\rho_i$ where $i \in \{\leq 1, \text{1-4}, \text{5-14}, \text{15-24}, \ldots, \text{75-84}, \text{85+}\}$. Let $P_{stri}$ be the resident population indexed by county, year, race and age group. The corresponding expected number of overdose deaths for county $s$, year $t$, race $r$ and age group $i$ is

$$E_{stri} = \rho_i P_{stri}. \tag{2}$$

Therefore the total expected number of overdose deaths $E_{tr}$ for year $t$ and race $r$, based upon the age-specific overdose death rate in the 2010 Ohio population, is

$$E_{tr} = \sum_{s,i} E_{stri}. \tag{3}$$

It follows that $\lambda_{tr}$ represents the state-level relative risk of overdose death for race $r$ in year $t$ compared to the corresponding risk for race $r$ in year $t$ expected from the age-specific overdose death rate in the 2010 Ohio population, commonly referred to as the standardized mortality ratio (SMR). Using the log link, we model the SMR as

$$\log(\lambda_{tr}) = \mathbf{X}_t{}'\boldsymbol{\beta}_r + \epsilon_{tr} \tag{4}$$

where $\boldsymbol{\beta}_r$ is a vector of race-specific fixed effects corresponding to the vector of covariates $\mathbf{X}_t$ containing an intercept, years since 2010 (i.e. $t - 2010$), an indicator of race (with White as the reference level) and the interaction of years since 2010 and race. We also include a term for the remaining race-specific heterogeneity $\epsilon_{tr}$ to capture variation not accounted for by the fixed effects. We assume an auto-regression of order 1 structure to account for temporal auto-correlation. Thus, we assume

$$\begin{cases} \epsilon_{tr} \overset{\text{ind}}{\sim} N(0, \sigma_r^2) & \text{for } t = 2010 \\ \epsilon_{tr}|\epsilon_{(t-1)r} \overset{\text{ind}}{\sim} N\left(\phi_r \epsilon_{(t-1)r}, \sigma_r^2\right) & \text{for } t > 2010 \end{cases} \tag{5}$$

where $\phi_r$ is a race-specific auto-regressive parameter and $\sigma_r^2$ is a variance.

The fixed effects are assigned independent flat priors. Auto-regressive parameters $\phi_r$ are assumed to be uniformly distributed over (0,1). All variance

parameters are assigned independent inverse gamma distributions with shape and scale parameters both equal to 0.5. The models were fit using Markov chain Monte Carlo implemented in NIMBLE [1] in R. The algorithm was run for 1,000,000 iterations, discarding the first 500,000 as burn-in, and then thinned by keeping every $100^{th}$ iteration. We assessed convergence visually using trace plots.

### 1.2 Spatio-temporal model

For a detailed examination of the county-level overdose death count data, we modify the multivariate Poisson linear mixed effects model introduced by Kline, Pan, and Hepler (2021) [2] to fit our current problem setting. This model complements the state-level analysis by providing insights into county-level overdose death patterns. It assumes that

$$Y_{str}|\lambda_{str} \overset{\text{ind}}{\sim} \text{Poisson}(\text{E}_{str}\lambda_{str}) \tag{6}$$

where $Y_{str}$ is number of overdose deaths for county $s$, year $t$ and race $r$ and $E_{str}$ is the corresponding expected number of overdose deaths for county $s$, year $t$ and race $r$ based upon the age-specific overdose death rate in the 2010 Ohio population. As in the time-series analysis, this formulation assumes the expected overdose rates between racial groups are homogeneous.

$E_{str}$ is defined in an analogous manner to $E_{tr}$ in the time-series model, but for the county-level. Follow the procedure detailed in the time series model and obtain $E_{stri}$ by summing over the age groups:

$$E_{str} = \sum_i E_{stri}. \tag{7}$$

It follows that $\lambda_{str}$ represents the relative risk of overdose death for race $r$ in county $s$ and year $t$ compared to the corresponding risk for race $r$ in county $s$ and year $t$ expected from the age-specific death rate in the 2010 Ohio population. Using the log link, we model the SMR as

$$\log(\lambda_{str}) = \mathbf{W}'_{st}\gamma_r + \delta_r\nu_{st} + \epsilon_{str} \tag{8}$$

where $\boldsymbol{\gamma}_r$ is a vector of race-specific fixed effects corresponding to the vector of covariates $\mathbf{W}_{st}$ containing an intercept and years since 2010. We also include a spatio-temporal shared component $\nu_{st}$ with race-specific loading $\delta_r$ and a term for the remaining race-specific heterogeneity $\epsilon_{str}$. For identification, we assume the loading for White Ohioans is equal to 1 (i.e. $\delta_0 = 1$).

Since racial groups within a county share a common environment, we include a shared component $\nu_{st}$ in the model to reflect this. We also use this term to account for spatial structure with an intrinsic conditional auto-regressive model and shared temporal auto-correlation using an auto-regression of order 1. That

is, we assume the following conditional distributions:

$$\begin{cases} \nu_{st}|\nu_{-st} \sim N\left(\frac{1}{w_{s+}}\sum_\ell w_{s\ell}\nu_{\ell t}, \frac{\tau^2}{w_{s+}}\right) & \text{for } t = 2010 \\ \nu_{st}|\nu_{-st}, \nu_{s(t-1)} \sim N\left(\eta\nu_{s(t-1)} + \frac{1}{w_{s+}}\sum_\ell w_{s\ell}(\nu_{\ell t} - \eta\nu_{\ell(t-1)}), \frac{\tau^2}{w_{s+}}\right) & \text{for } t > 2010 \end{cases} \tag{9}$$

where $\eta$ is a temporal auto-regressive parameter, $w_{s\ell}$ is an indicator of whether counties $s$ and $\ell$ are neighbors as defined by adjacency, $w_{s+}$ is the number of neighbors of county $s$, and $\tau^2$ is a variance parameter. We constrain $\sum_s \nu_{st} = 0$ for every year $t$ which allows us to use equation (9) as a valid process model [3].

Acknowledging that racial groups may experience the same environment differently, we include a race-specific loading $\delta_r$ to allow for differing effects of the shared component by race. We also model race-specific variation $\epsilon_{str}$ to capture variation not accounted for by the shared component or fixed effects. We assume an auto-regression of order 1 structure to account for temporal auto-correlation within a county. Thus, we assume

$$\begin{cases} \epsilon_{str} \overset{\text{ind}}{\sim} N(0, \omega_r^2) & \text{for } t = 2010 \\ \epsilon_{str}|\epsilon_{s(t-1)r} \overset{\text{ind}}{\sim} N\left(\theta_r\epsilon_{s(t-1)r}, \omega_r^2\right) & \text{for } t > 2010 \end{cases} \tag{10}$$

where $\theta_r$ is a race-specific auto-regressive parameter and $\omega_r^2$ is a variance.

The fixed effects and $\delta_r$ are assigned independent flat priors. Auto-regressive parameters $\eta$ and $\theta_r$ are assumed to be uniformly distributed over (0,1). All variance parameters are assigned independent inverse gamma distributions with shape and scale parameters both equal to 0.5. The models were fit using Markov chain Monte Carlo implemented in NIMBLE in R. For the spatio-temporal model the algorithm was run for 1,000,000 iterations, discarding the first 500,000 as burn-in, and then thinned by keeping every $100^{th}$ iteration. We assessed convergence visually using trace plots.

## References


[1] de Valpine, P., D. Turek, C.J. Paciorek, C. Anderson-Bergman, D. Temple Lang, and R. Bodik. *Programming with models: writing statistical algorithms for general model structures with NIMBLE.* Journal of Computational and Graphical Statistics. 2017. 26: 403-413.

[2] Kline D, Pan Y, Hepler SA. *Spatiotemporal Trends in Opioid Overdose Deaths by Race for Counties in Ohio.* Epidemiology. 2021 Mar 1;32(2):295-302.

[3] Banerjee S, Carlin BP, Gelfand AE. *Hierarchical Modeling and Analysis for Spatial Data.* Boca Raton, FL: Chapman & Hall/CRC; 2004.
