# Supplementary material for: The impact of fentanyl on state- and county-level psychostimulant and cocaine overdose death rates by race in Ohio from 2010 to 2020: a time series and spatiotemporal analysis
Source: Harm Reduct J. 2024 Jan 17;21:13. doi: 10.1186/s12954-024-00936-9 (PMC10792830; doi:10.1186/s12954-024-00936-9)
Supplement: Supplementary file 2 — Additional file 2. County-level maps of Ohio, including Black versus White rate ratio estimates of cocaine and psychostimulant-involved SMRs from 2010-2020, and a table of spatiotemporal cocaine and psychostimulant-involved overdose rate ratios by annual change and race. [file 12954_2024_936_MOESM2_ESM.docx]

**Supplement 2**

**Figure S1.** County-level map of Ohio with pertinent cities for reader’s reference


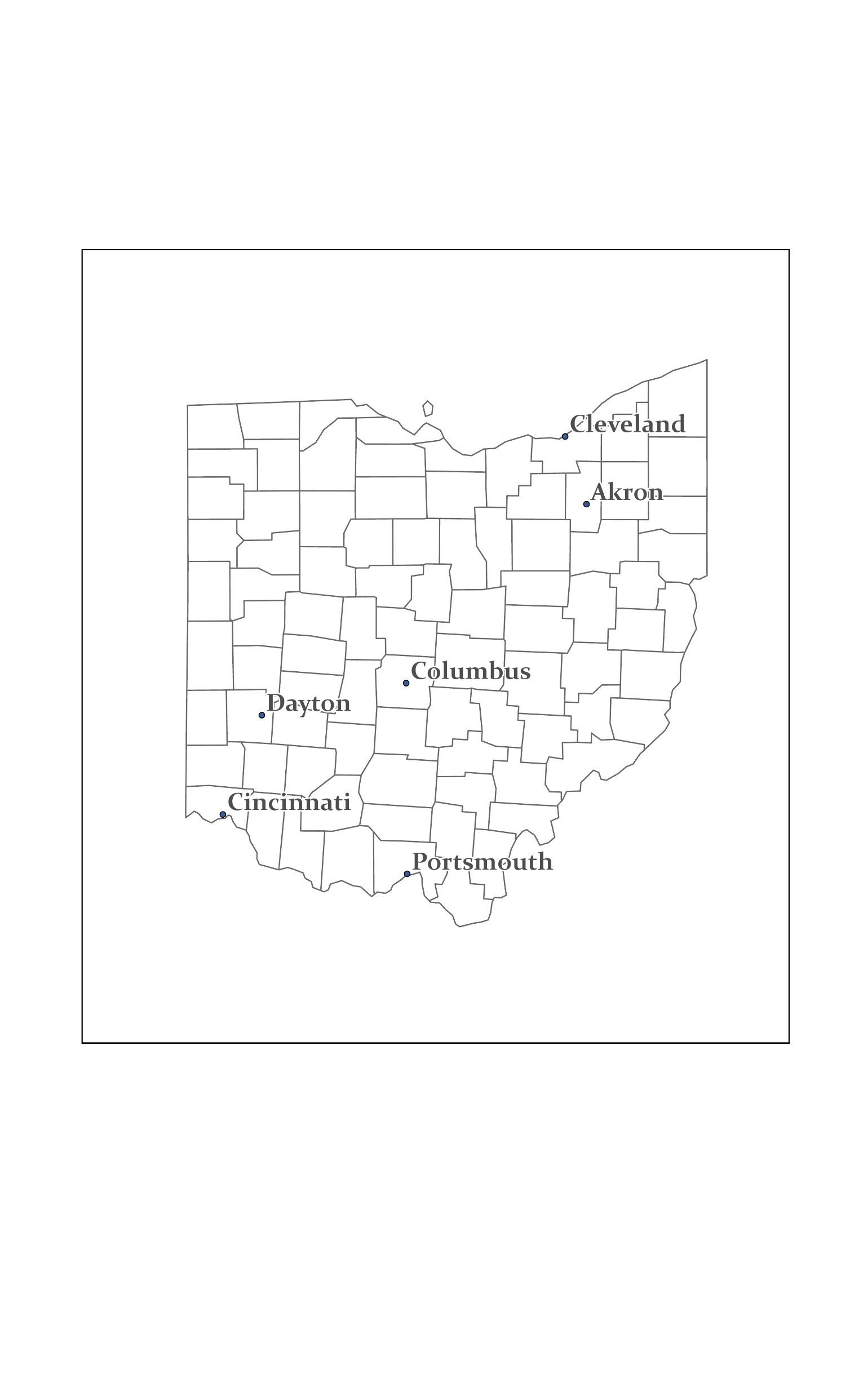


**Figure S2**. Black versus White Rate Ratio estimates of cocaine-involved SMRs, 2010-2020


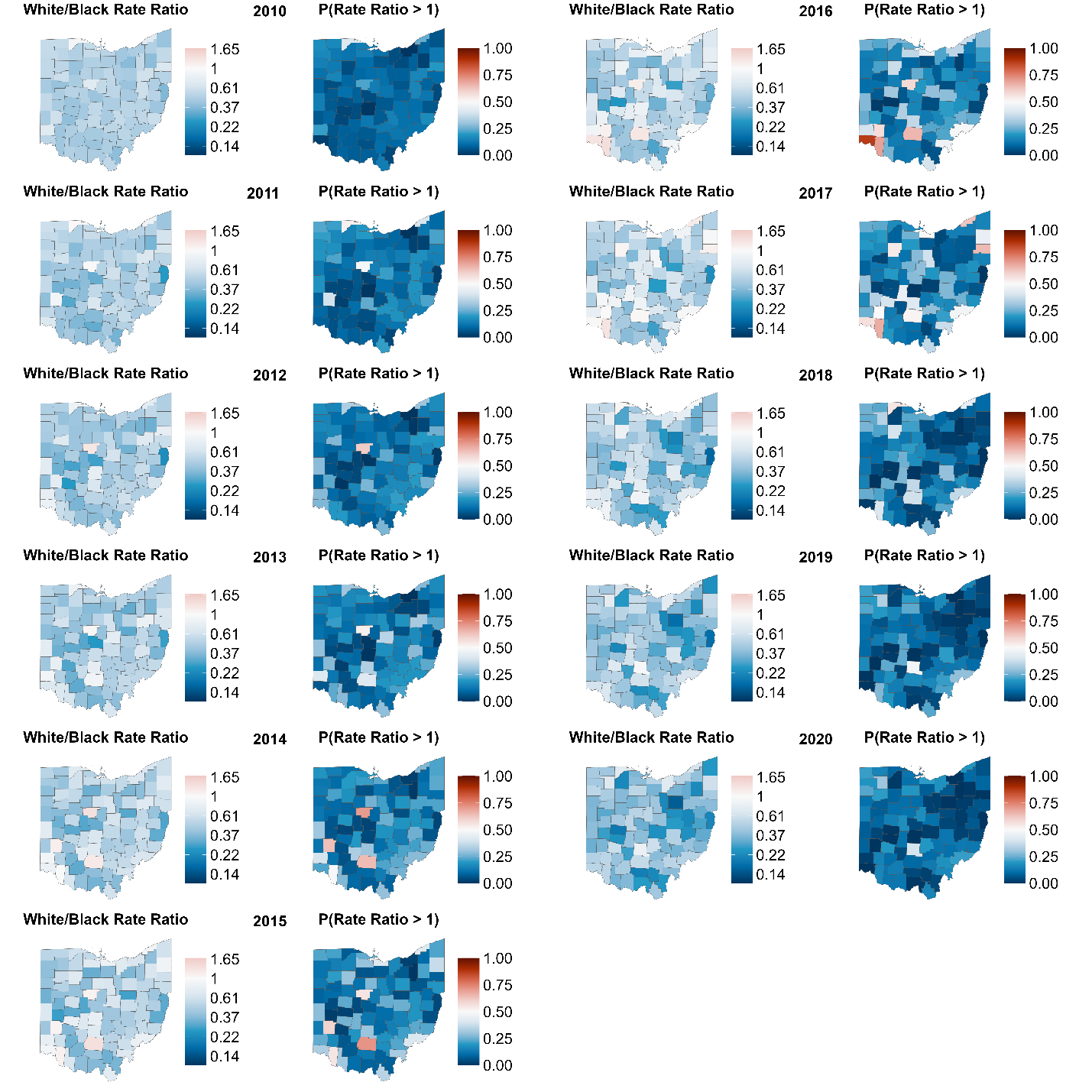


**Figure S3**. Black versus White Rate Ratio estimates of psychostimulant-involved SMRs, 2010-2020


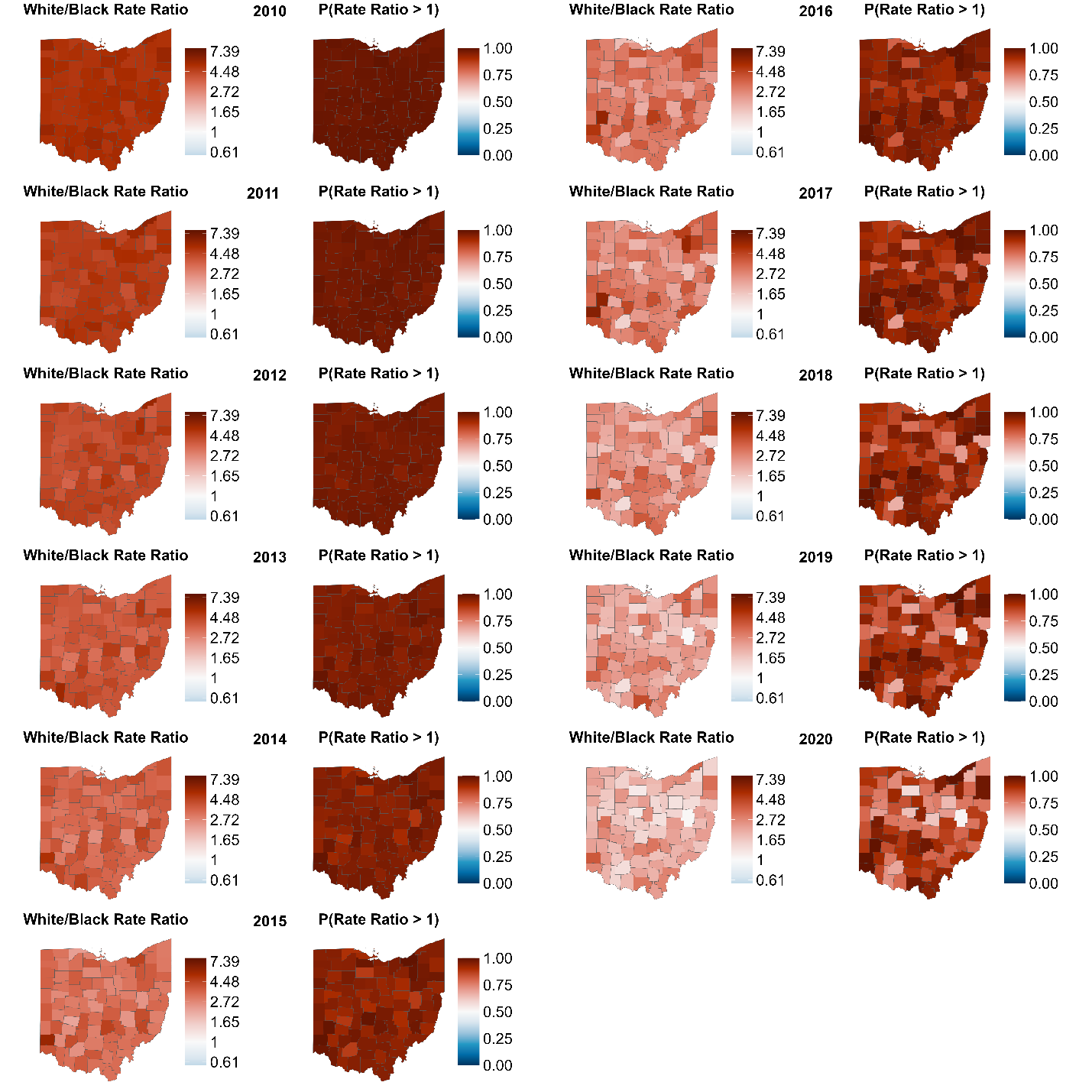


**Figure S4.** Cocaine-involved SMR by county and race, 2010-2020


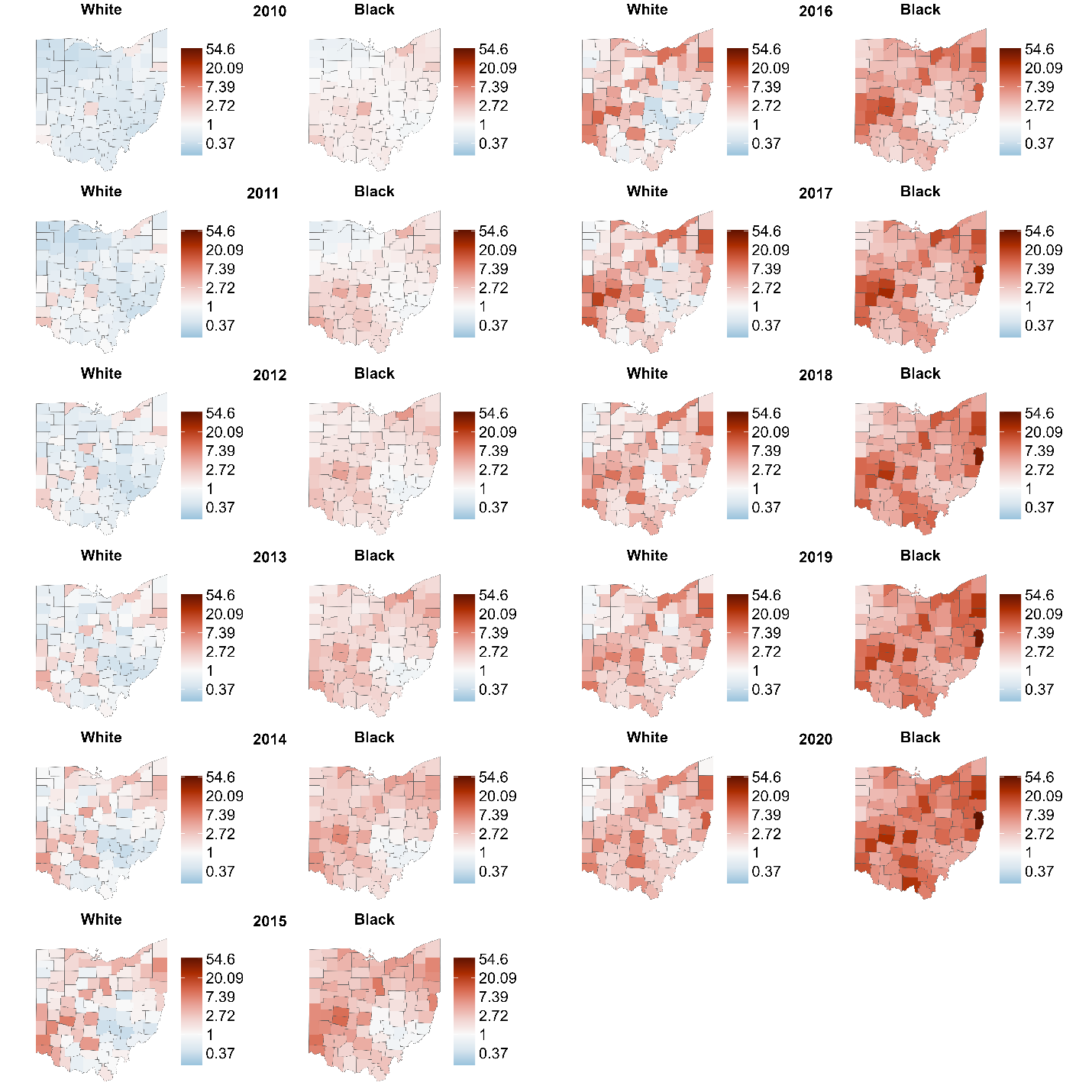


**Figure S5.** Psychostimulant-involved SMR by county and race, 2010-2020


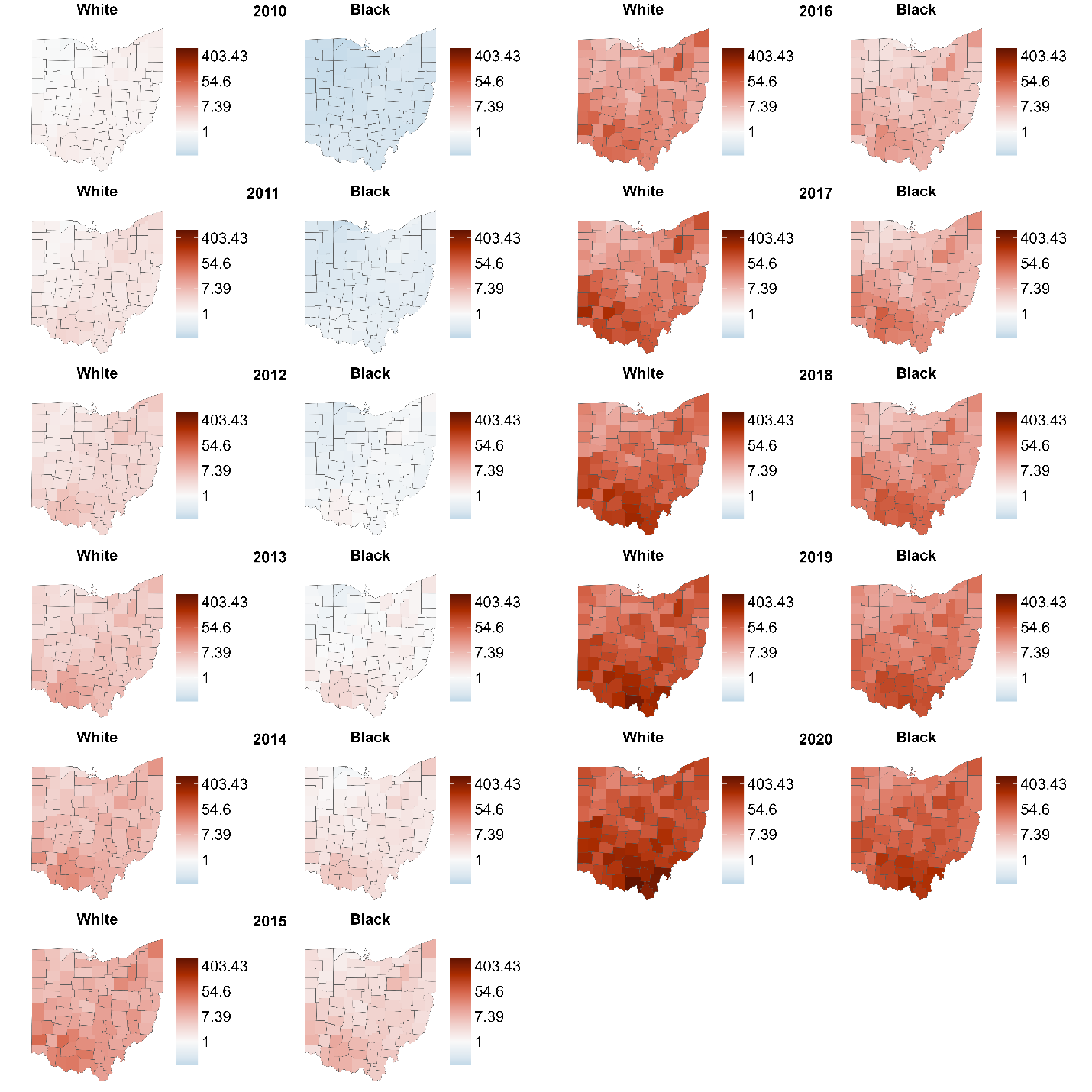


**Figure S6**. Posterior probability that the shared component ($\nu_{st})$is greater than 0 in the spatial model of all cocaine overdose deaths, 2010-2020
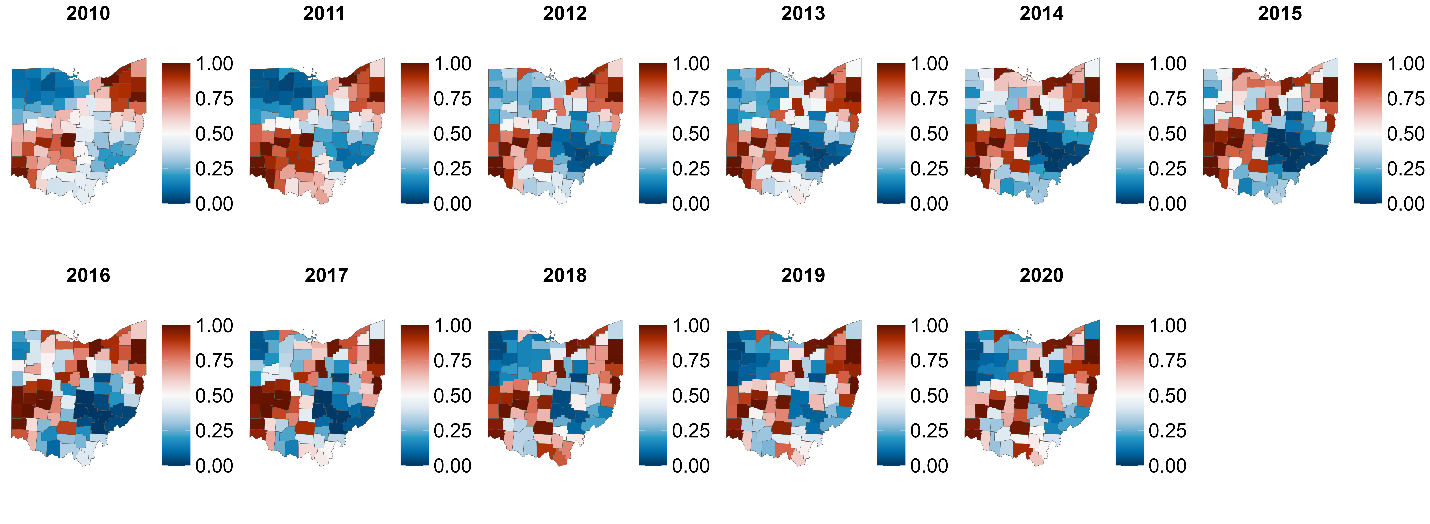


**Figure S7**. Posterior probability that the shared component ($\nu_{st})$is greater than 0 in the spatial model of all psychostimulant overdose deaths, 2010-2020


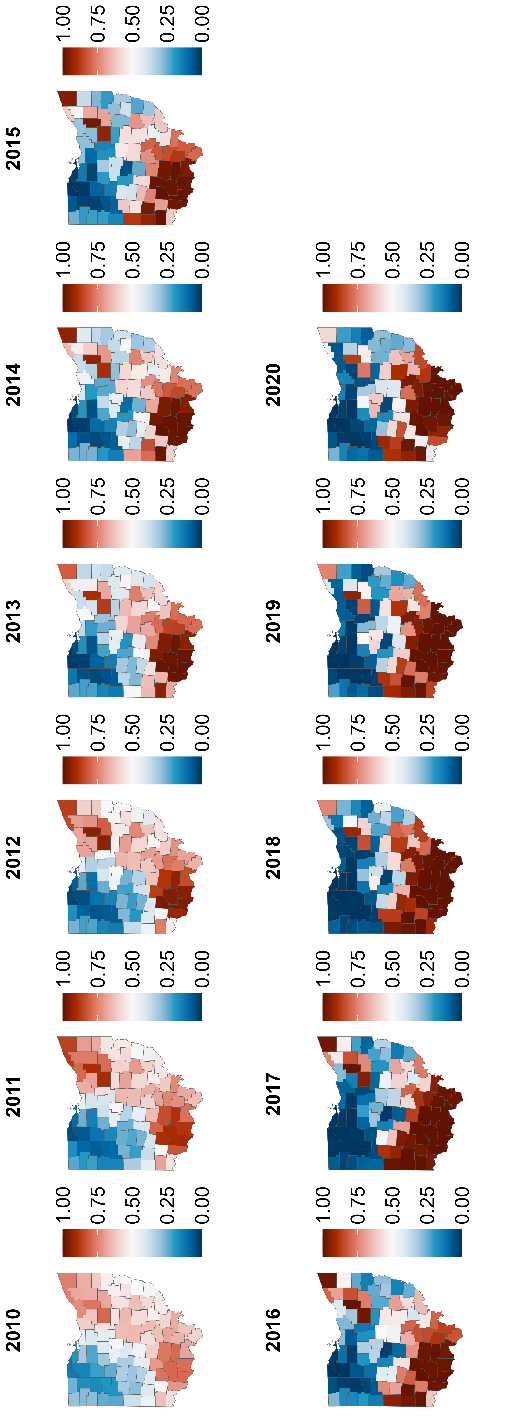


**Table S1.** Spatio-temporal cocaine and psychostimulant-involved overdose rate ratio by annual change and race, Ohio 2010-2020

|  | **Time (1 year change)** | | **Race Comparison** | |
| --- | --- | --- | --- | --- |
|  | **RR^1^** | **95% CrI^1^** | **RR^1^** | **95% CrI** |
| **All cocaine overdoses** | | | | |
| White | 1.18 | (1.15, 1.21) | ref | - |
| Black | 1.20 | (1.16, 1.25) | 1.02 | (0.98, 1.07) |
| **All psychostimulant overdoses** | | | | |
| White | 1.57 | (1.52, 1.61) | ref | - |
| Black | 1.73 | (1.56, 1.93) | 1.10 | (0.99, 1.23) |
| **Cocaine overdoses not involving fentanyl** | | | | |
| White | 1.00 | (0.97, 1.03) | ref | - |
| Black | 1.05 | (1.00, 1.09) | 1.05 | (1.00, 1.09) |
| **Psychostimulant-involved overdoses excluding fentanyl** | | | | |
| White | 1.33 | (1.28, 1.37) | ref | - |
| Black | 1.50 | (1.31, 1.73) | 1.13 | (0.99, 1.31) |
| *1. CrI= Credible Interval; RR=Rate Ratio* | | | | |
